# Study on immobilization of composite pollutants in antimony mining areas by target-enriched sulfate-reducing bacteria from antimony tailings

**DOI:** 10.3389/fmicb.2025.1676884

**Published:** 2025-11-25

**Authors:** Xuezhe Zhu, Yupin Zhou, Mingjiang Zhang, Xiao Yan, Xingyu Liu, Zhihe Dou

**Affiliations:** 1 National Engineering Research Center for Environment-friendly Metallurgy in Producing Premium Non-ferrous Metals, China GRINM Group Co., Ltd., Beijing, China; 2 School of Metallurgy, Northeastern University, Shenyang, China; 3 GRINM Resources and Environment Tech. Co., Ltd, Beijing, China; 4 General Research Institute for Nonferrous Metals, Beijing, China; 5 Beijing Engineering Research Center of Strategic Nonferrous Metals Green Manufacturing Technology, Beijing, China; 6 GRIMAT Engineering Institute Co., Ltd., Beijing, China; 7 Guobiao (Beijing) Testing & Certification Co., Ltd., China GRINM Group Co., Ltd., Beijing, China; 8 Institute of Earth Science, China University of Geosciences (Beijing), Beijing, China

**Keywords:** sulfate-reducing bacteria, antimony mining area, nutrient factor optimization, composite pollution, bioremediation

## Abstract

Remediating composite pollution in antimony mining areas, characterized by coexisting anionic metalloids (Sb, As) and cationic heavy metals (e.g., Pb, Cd, Cu, Zn), remains a significant challenge. This study demonstrates the effective immobilization of these complex pollutants using target-enriched sulfate-reducing bacteria (SRB) in heavy metal pollution remediation research. Indigenous SRB were enriched from antimony tailings and their application in remediating typical composite-polluted wastewater and tailings in antimony mining areas. The SRB community enriched in modified Postgate medium was dominated by the genus *Clostridium*, confirming the successful enrichment of functional sulfate-reducing microorganisms. Results of nutrient optimization showed that the optimal nutrients were 2.0 mL/L sodium lactate (carbon source), 1.2 g/L yeast extract (nitrogen source), and 0.5 g/L K_2_HPO_4_·3H_2_O (phosphorus source), and the corresponding medium enhanced SRB activity, increasing the Fe^2+^ immobilization rate to >90%. In simulated wastewater from antimony mining areas, SRB effectively immobilized various pollutants with immobilization rates of 97.9% for Sb, 82.8% for As, 91.7% for Pb, 99.7% for Cd, 99.5% for Cu, and 99.8% for Zn. Characterization via SEM-EDS, XRD, and FT-IR revealed that immobilization products were primarily composed of heavy metal sulfides, carbonates, hydroxides, and microbial extracellular polymeric substances (EPS). Biological tissues were also involved in the heavy metal immobilization process. For antimony tailings, Zn was effectively remediated whereas Sb and As exhibited significant redissolution, which was significantly suppressed by reducing sulfate concentration. This study provides valuable insights into managing complex pollution involving coexisting metalloids (Sb, As) and heavy metals (Pb, Cd, Cu, Zn).

## 1 Introduction

Antimony (Sb), a strategic nonferrous metal, is associated with mining activities that have caused severe heavy metal(loid)s contamination in mine waters and tailings (Fu et al., 2010; He et al., 2012). Such polluted sites are dominated by composite pollution, including antimony (Sb), arsenic (As), lead (Pb), cadmium (Cd), copper (Cu), and zinc (Zn), etc. (Qi et al., 2022; Luo et al., 2024; Cakmak et al., 2025). These heavy metal(loid)s pose significant risks to ecological systems and human health due to their inherent toxicity, high potential for bioaccumulation, and environmental persistence (Liu et al., 2022; Xie and Ren, 2022). For instance, Sb exposure is linked to respiratory diseases (Su et al., 2022) and dermatological damage (Chen et al., 2023), whereas As is a well-established carcinogen that adversely affects multiple organ systems (Balali-Mood et al., 2021; Li et al., 2021). The improper disposal of antimony-laden wastewater and tailings has resulted in extensive contamination of adjacent soils, groundwater, and surface water (Luo et al., 2021; Nguyen et al., 2021). This situation underscores the urgent need to develop adaptable remediation technologies capable of addressing a broad spectrum of contaminants. Current strategies encompass a range of physical–chemical and biological approaches, including the creation of activated materials from waste (Zeng et al., 2025), electrokinetic processes for resource enrichment (Lan et al., 2025), and bioremediation using functional microorganisms.

Bioremediation, particularly utilizing sulfate-reducing bacteria (SRB), has emerged as a promising strategy for rehabilitating heavy metal-contaminated environments, owing to its cost-effectiveness and environmental compatibility. SRB are anaerobic microorganisms that derive energy by reducing sulfate (SO_4_^2−^) to sulfide (S^2−^) under anaerobic conditions. The generated S^2−^ can react with heavy metal ions to form insoluble metal sulfide precipitates (Diez-Ercilla et al., 2014; Dong et al., 2024), which exhibit significantly lower toxicity and mobility compared to their ionic forms. Furthermore, SRB contribute to heavy metal immobilization through additional mechanisms, including bioaccumulation (Cetin et al., 2008; Kiran et al., 2017), biosorption (Yue et al., 2015), and the precipitation of metal hydroxides or carbonates facilitated by the alkaline conditions created through their metabolic activity (Bai et al., 2023). The efficacy of SRB in remediating heavy metal-contaminated sites and treating heavy metal-laden wastewater has been well-documented in numerous studies. For example, SRB has been successfully applied to treat wastewater containing high concentrations of Cd, Pb, and Zn with an immobilization rate of more than 90% (Zhang Z. et al., 2022b). Similarly, other studies have shown effective removal of Cu, Ni, and Zn from acid mine drainage using SRB-based bioreactors (Mafane et al., 2025), and high-efficiency immobilization of Cd and Pb by specific SRB strains like *Desulfovibrio desulfuricans* (Chen et al., 2025).

However, the application of SRB for remediating antimony mining areas faces two specific and significant challenges. (1) SRB strains isolated from common or low-concentration environments often exhibit poor adaptability to the high-concentration, complex pollution typical of antimony mines (Zampieri et al., 2022; Zhang et al., 2023); (2) The unique chemistries of Sb and As allow them to form soluble thio-complexes (e.g., SbS_3_^3−^, AsS_3_^3−^) with sulfides, leading to the re-solubilization of initially immobilized products and making their stable sequestration particularly challenging (Han et al., 2018). Consequently, constructing an effective SRB remediation system for the composite pollution in antimony mining areas remains a critical and unresolved task, as most previous studies have focused on single or cationic metals without adequately addressing the complexities posed by co-existing anionic metalloids.

The efficacy of SRB-based bioremediation is highly dependent on the composition of the medium. The Postgate medium is commonly employed for the isolation and cultivation of SRB; however, it often requires optimization to effectively support SRB growth and metabolic activity in specific environmental contexts (Wolicka and Jarzynowska, 2012; Labastida et al., 2024). Key parameters that affect SRB performance include the type and concentration of carbon, nitrogen and phosphorus sources. For instance, lactate is a well-established carbon source for many SRB species, serving as an effective electron donor for sulfate reduction (Teclu et al., 2009; Zhang et al., 2016). However, its optimal concentration is strain-dependent and must be determined for the specific application (Zhao et al., 2023). The traditional postgate medium consists of an organic nitrogen source, yeast extract, and an inorganic nitrogen source, NH_4_Cl, and the phosphorus source is often chosen to be dipotassium hydrogen phosphate, but its optimal concentration varies with the scenario in which it is used. Therefore the optimization of the medium is a necessary process for SRB remediation of specific polluted environments, and there is a significant lack of research on the optimization of the medium for SRB obtained from enrichment in the special environment of antimony mining areas.

Additionally, sulfate concentration is closely related to the abundance and activity of SRB, while the amount of S^2−^ produced is critical for the immobilization and re-solubilization of Sb and As. In a study on arsenic-contaminated rice paddy soil, the exogenous addition of sulfate significantly enhanced the abundance of SRB and decreased the concentrations of As(III) and total arsenic; however, this effect was absent when sulfate reduction was chemically inhibited (Xu et al., 2019). Conversely, other studies have reported that the input of organic matter (Yan et al., 2020) and sulfate (Yang et al., 2022; Luo et al., 2025) can mobilize arsenic, leading to a significant increase in its groundwater concentration. Therefore, the specific conditions that promote the effective immobilization of Sb and As, enabling synergistic remediation alongside cationic heavy metals in composite pollution scenarios, remain inadequately explored.

Most previous SRB-based remediation studies have focused on single or cationic heavy metal pollutants, often using strains from common environments. However, their application is limited for the complex, high-concentration composite pollution found in antimony mining areas. This study addresses two key gaps: (1) the enrichment and application of indigenous SRB consortia from the challenging environment of antimony tailings, ensuring better adaptability, and (2) a systematic investigation into their efficacy for the simultaneous immobilization of a composite mixture of anionic metalloids (Sb, As) and cationic heavy metals, which represents a more realistic and complex remediation scenario.

The main objectives of this study were: (1) to isolate and enrich indigenous SRB from antimony tailings using a modified Postgate medium; (2) to optimize the culture conditions for the enriched SRB, including the concentrations of carbon, nitrogen, and phosphorus sources; (3) to evaluate the performance and characterize the immobilization products of the optimized SRB consortium in remediating simulated heavy metal-polluted wastewater; (4) to assess the effectiveness of the optimized SRB in remediating actual antimony tailings and to investigate the critical role of sulfate concentration in the remediation process, with a special focus on controlling the immobilization and re-solubilization of Sb and As. This study focuses on the targeted enrichment and application of SRB from the specific environment of antimony mine pollution. These findings are intended to provide valuable insights for the development of effective SRB-based bioremediation strategies for complex heavy metal-contaminated mining environments.

## 2 Materials and methods

### 2.1 Sample collection and preparation

Antimony tailings samples used in the experiments were collected from a typical antimony mining area in Guangxi Zhuang Autonomous Region, and the samples were observed to be in the form of gray-black powder. The samples used for microbial enrichment were sealed and stored, and then mixed and used directly when enriching microorganisms. The experimental samples used for remediation of heavy metal pollution were air-dried, pulverized and passed through a 20-mesh sieve before mixing and use.

The agents used in the experiments were analytically pure and the water used was deionized water.

### 2.2 Enrichment of sulfate-reducing functional microorganisms

The antimony tail samples were inoculated into modified Postgate medium at 5% (wt/v, g/mL). The composition of the modified Postgate medium is shown in Table 1. Inoculated media were transferred to 400-mL conical flasks, sealed with butyl rubber stoppers to ensure anaerobic conditions, and incubated in a thermostatic incubator at 30 °C. The appearance of black precipitate in the medium was used as a sign of successful sulfate reduction. After observation of black precipitate, 5% (v/v) inoculum was transferred to fresh modified Postgate medium to pass on the culture. This passaging culture process was repeated several times to enrich the SRB community and enhance its metabolic activity. The enrichment effect was assessed by monitoring changes in Fe^2+^ concentration, pH, and oxidation reduction potential (ORP) of the passaged cultures. Triplicate samples were prepared for each experimental group to ensure the reliability of the results, and the subsequent optimization and repair experiments were similarly conducted in triplicate.

**Table 1:** Composition of the modified postgate medium.

**Reagent**	**Concentration (g/L)**
MgSO_4_·7H_2_O	4.0
Sodium lactate ^1^	1.0
Yeast extract	1.2
Na_2_SO_4_	0.5
K_2_HPO_4_·3H_2_O	0.5
CaCl_2_·2H_2_O	0.1

Sodium lactate (50–60% aqueous solution) was added at a volume concentration (mL/L).

### 2.3 Studies on the effects of nutritional factors

#### 2.3.1 Experimental design

To optimize the culture conditions for the enriched SRB, a series of single-factor experiments were designed, each targeting one of the four nutrient factors: carbon source (sodium lactate), organic nitrogen source (yeast extract, YE), inorganic nitrogen source (ammonium chloride, NH_4_Cl), and phosphorus source (KH_2_PO_4_ or K_2_HPO_4_·3H_2_O), to assess their individual effects on SRB activity. The enriched SRB cultures were inoculated at 5% (v/v) into modified Postgate medium, where only the concentration of the target nutrient was varied while all other components remained constant. Specific concentration settings for each factor are provided in Table 2. After preparing media with the specified compositions, cultures were incubated at 30 °C for 7 days. Measurements of Fe^2+^ immobilization rate, total sulfur concentration, pH, and ORP were then performed to determine the optimal type and concentration for the carbon, nitrogen, and phosphorus sources.

**Table 2:** Concentration settings for each nutrient factor.

**Nutrient factors**	**Concentration (g/L)**
Carbon sources	0, 1.0, 2.0, 3.0, 4.0
Organic nitrogen sources	0, 0.6, 1.2, 1.8, 2.4
Inorganic nitrogen sources	0, 0.5, 1.0, 1.5, 2.0
Phosphorus sources	P1(K_2_HPO_4_·3H_2_O, 0.25), P2(K_2_HPO_4_·3H_2_O, 0.50), P3(KH_2_PO_4_, 0.25), P4(KH_2_PO_4_, 0.50)

#### 2.3.2 Growth kinetics of SRB after optimization of nutrient factors

The growth kinetics of SRB was studied under the optimal conditions determined by the nutrient factor impact experiment. Optical density at 600 nm (OD_600_) was measured daily for 16 days using a spectrophotometer (Shimadzu UV-1800). During this period, changes in total Fe concentration, pH and ORP were also monitored to understand the relationship between microbial growth and metabolic activity.

### 2.4 Bioremediation of heavy metal polluted wastewater from antimony mine by SRB

#### 2.4.1 Preparation of simulated wastewater

A simulated heavy metal wastewater was prepared based on the composition of antimony tailings leachate. The concentrations of heavy metals in the simulated wastewater were based on the average values measured in actual tailings leachate samples, as shown in Table 3. The pH of the wastewater was adjusted to 6.5 ± 0.1 using 0.1 mol/L HCl or NaOH before use.

**Table 3:** Concentrations of various heavy metal elements in the simulated wastewater.

Element	Sb	As	Pb	Cd	Cu	Zn
Concentration (mg/L)	11.52 ± 0.12	4.82 ± 0.30	0.36 ± 0.08	6.17 ± 0.04	3.78 ± 0.04	10.24 ± 0.30

#### 2.4.2 Wastewater treatment experiments

In this study, batch treatment was used to set up a control group CK and a remediation group SRB, respectively. The same amount of heavy metal concentrate was added to the control and remediation groups to simulate the polluted wastewater from actual antimony mining area. The corresponding medium and SRB bacterial solution were added to the remediation group with an inoculum of 5%, while equal amounts of deionized water were added to the blank group. The cultures were sealed and incubated in a constant temperature incubator at 30 °C for 7 days. At the end of the remediation period, the cultures were centrifuged, the supernatant was collected, and the heavy metal concentrations were measured using ICP-OES. The immobilization rate of each heavy metal was calculated using the formula:


$$\text{Immobilization Rate}\;(\%)=\frac{c_{\text{initial}}-c_{\text{final}}}{c_{\text{initial}}}\times 100\%$$

#### 2.4.3 Characterization of immobilization products

The precipitated products were collected by centrifugation and incubated in an ultra-low-temperature refrigerator at −80 °C (TDE30086FV-ULTS. Thermo Scientific, USA) to solid state and then freeze-dried in a vacuum freeze-dryer (Labconco™ FreeZone™ 2.5 L, ThermoFisher, USA). Subsequently, they were sealed and stored and subjected to scanning electron microscopy (SEM) (Hitachi SU8010, Japan), X-ray diffraction (XRD) (SmartLab, Rigaku, Japan) and Fourier transform infrared spectroscopy (FT-IR) (IS50, NicoletiS50, Thermo Fisher, USA) characterization to determine the elemental composition of the immobilized products and the immobilization mechanism.

### 2.5 Bioremediation of antimony tailings by SRB

#### 2.5.1 Study on the change of heavy metal concentration of tailings during the bioremediation process with the remediation time

The strains in logarithmic phase were inoculated into the prepared medium at a ratio of 5%, and antimony tailings were mixed with the optimized SRB cultures in 300 mL specification serum bottles at a solid–liquid ratio of 1:10 (w/v), and sealed and cultivated in a constant temperature incubator at 30 °C. Sterile water was used instead of SRB culture for control experiments. The mixture was incubated at 30 °C for 60 days and samples were taken periodically using a sterile syringe to monitor the concentration of contaminants in the supernatant as well as changes in pH and ORP values.

#### 2.5.2 Research on the effect of sulfate concentration on the bioremediation effect of antimony tailings

In order to study the effect of sulfate concentration on the remediation of antimony tailings, different amounts of magnesium sulfate (MgSO_4_·7H_2_O) were added to the tailings-SRB system. Four groups of treatments were designed: CK (blank control), M1 (SRB + 1.0 g/L MgSO_4_·7H_2_O), M2 (SRB + 2.0 g/L MgSO_4_·7H_2_O), and M3 (SRB + 4.0 g/L MgSO_4_·7H_2_O, original concentration). To ensure consistent magnesium ion concentrations among all treatments, the same amount of MgCl_2_ was added to the M1 and M2 groups to match the magnesium concentration in the M3 group. Tailings were treated for 7d and 21d, and contaminant concentrations in the supernatant were analyzed to evaluate the effect of sulfate concentration on the remediation effect.

### 2.6 Analytical methods

#### 2.6.1 Analysis of metal and sulfur-phosphorus concentrations

Inductively coupled plasma emission spectrometry (ICP-OES, Agilent 725, Agilent Technologies Co. Ltd., USA) was used to determine the concentrations of (analogous) metallic and sulfurous elements in the incubation supernatant, simulated effluent, and tailings leachate. Prior to analysis, samples were filtered through a 0.22 μm needle filter to remove particles. Calibration curves were prepared using standard solutions of each element and quality control was ensured by analyzing blank and spiked samples for recovery validation. Detection limits were 0.01, 0.01, 0.01, 0.01, 0.01, 0.01, 0.10 mg/L for Sb, As, Cd, Pb, Cu, Zn, and S, respectively.

#### 2.6.2 pH and ORP measurements

pH and oxidation–reduction potential (ORP) were measured using an ion meter (PXSJ-227 L, INSEA, Shanghai) equipped with a pH composite electrode (E201-L, INSEA, Shanghai) and an ORP composite electrode (501, INSEA, Shanghai).

#### 2.6.3 Microbial community analysis

In order to characterize the microbial community structure during the enrichment process, microorganisms from the different passages of the culture supernatant were analyzed using the Power Soil DNA isolation kit (MoBio Laboratories) to extract DNA from tailings samples and enrichment cultures at different passaging culture stages. The V4 region of the 16S rRNA gene was amplified using primer set 338F (5′-ACTCCTACGGGGAGGCAGCAG-3′). The amplified products were sequenced on the Illumina MiSeq platform at a commercial sequencing facility (Majorbio, Shanghai, China). Sequencing data were processed using the QIIME 2 process.

### 2.7 Experimental design overview

**Figure 1:**
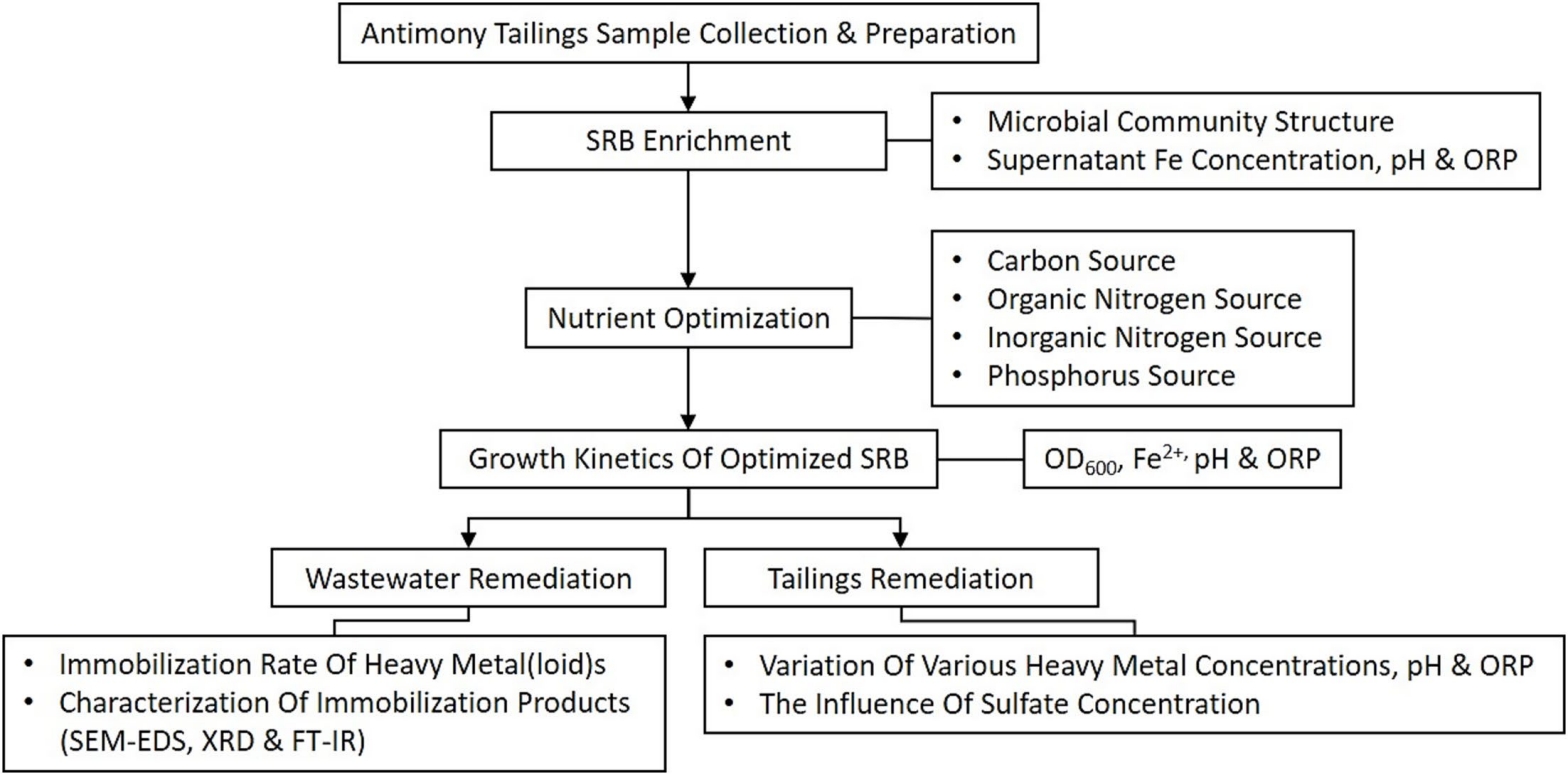
Schematic illustration of the experimental workflow for the enrichment, optimization, and application of sulfate-reducing bacteria (SRB) in remediating heavy metal pollution from antimony mining areas.

The overall experimental procedure is schematically illustrated in Figure 1. This study commenced with the collection of antimony tailings and the enrichment of indigenous sulfate-reducing bacteria (SRB). The enriched consortium was subsequently optimized by evaluating key nutrient factors and its growth kinetics. The performance of the optimized SRB was then assessed for the remediation of both simulated heavy metal wastewater and solid antimony tailings. The remediation efficacy was determined through heavy metal(loid) immobilization rates and the characterization of precipitation products, while the influence of sulfate concentration was specifically investigated in the tailings system.

## 3 Results and discussion

### 3.1 Enrichment of sulfate-reducing functional microorganisms

#### 3.1.1 Analysis of microbial community structure in the enrichment process

**Figure 2:**
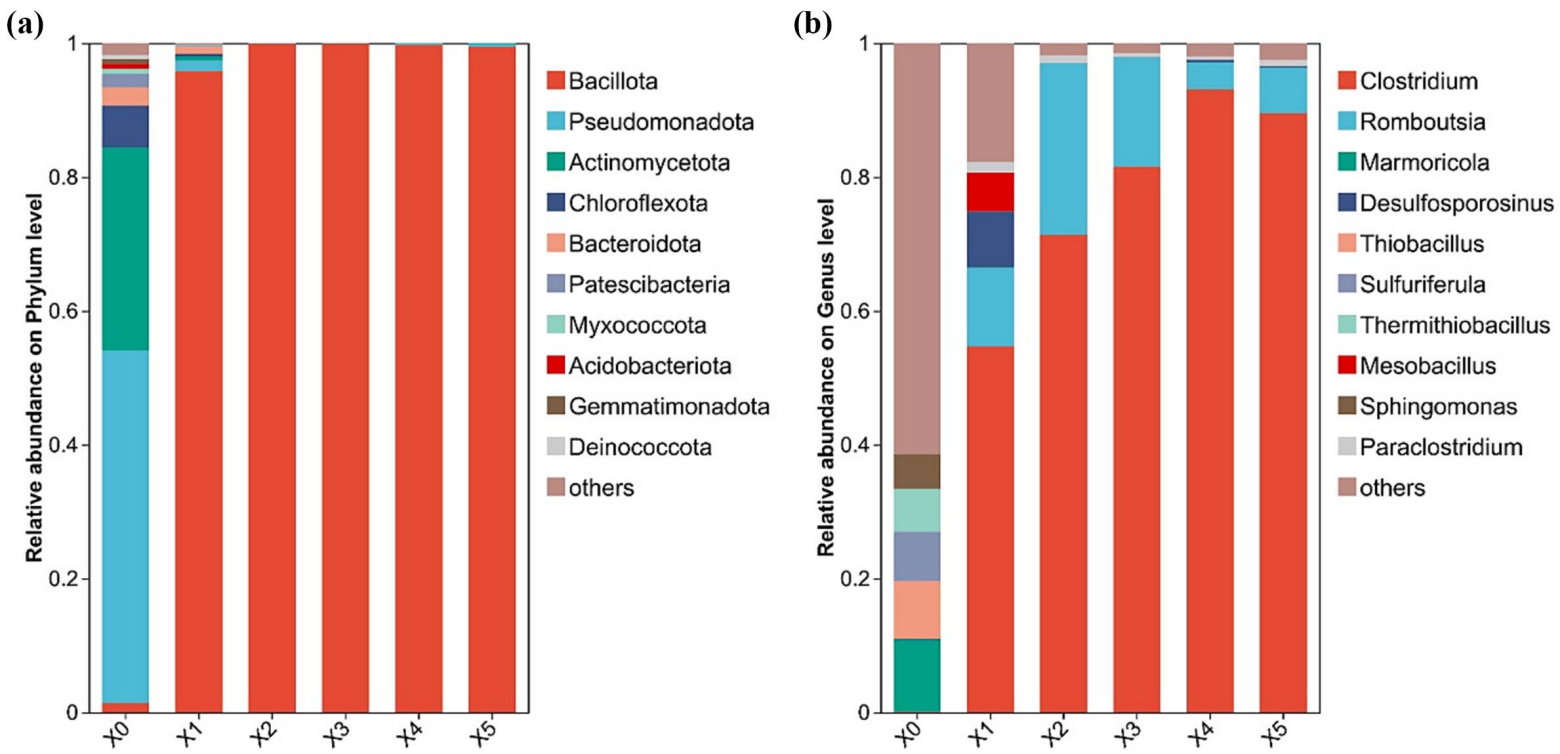
Changes in microbial community structure during the enrichment process **(a)** phylum level; **(b)** genus level.

High-throughput sequencing was employed to analyze the microbial communities throughout the enrichment process, revealing the dynamics of the community structure at both the phylum and genus levels (Figure 2). The phylum level analysis is shown in Figure 2a, from which it can be seen that before enrichment, four phyla, Pseudomonadota, Actinomycetota, Chloroflexota, and Bacteroidota, which are four important phyla in the bacterial domain, were dominant. They are four important phyla in the bacterial domain, which are characterized by diverse metabolism, antibiotic production, photosynthesis or adaptation to extreme environments, and degradation of complex carbohydrates, respectively, and are widely distributed in natural environments and living organisms, and play key roles in ecological cycles, human health, and industrial applications (Zheng et al., 2024). Whereas the abundance of these phyla decreased dramatically after enrichment, Bacillota became the dominant phylum after enrichment and accounted for more than 80% of the microbial community in late transfer. This is consistent with the known taxonomic affiliation of many SRBs, which belong to the phylum Bacillota (Sanchez-Andrea et al., 2013).

The genus-level microbial community structure is shown in Figure 2b. The initial microbial community exhibited higher diversity, with the top five genera (accounting for the highest abundance) being *Marmoricola, Thiobacillus, Sulfuriferula, Thermithiobacillus,* and *Sphingomonas*. These genera are associated with rock colonization or organic matter degradation, sulfur oxidative metabolism, sulfur-associated metabolism (possibly thermophilic), thermophilic sulfur oxidation, and broad-spectrum organic matter degradation or environmental remediation, respectively (Yang et al., 2015; Owczarek-Koscielniak and Sterflinger, 2018; Xu et al., 2023). Among them, *Thiobacillus, Sulfuriferula,* and *Thermithiobacillus* are capable of oxidizing metal sulfide ores, which can induce heavy metal leaching (Vardanyan and Vyrides, 2019). After one transfer, anaerobic *Clostridium* became the dominant genus with an abundance of 54.7%. After the first transfer, the anaerobic genus *Clostridium* became dominant, with an abundance of 54.7%. Its abundance increased with the number of transfers, reaching 93.1% and 89.5% after the fourth and fifth transfers, respectively. In contrast, the abundance of these five non-SRB genera, which were dominant in the initial culture, decreased to less than 1.0%. Notably, the abundance of genera responsible for metal sulfide ore oxidation declined significantly, suggesting that the enrichment process suppressed the growth of oxidizing bacteria. As reported in the literature, certain species of *Clostridium* possess sulfate-reducing capacity (Guo et al., 2025; Zhang et al., 2025), leading us to conclude that sulfate-reducing microorganisms were successfully enriched.

#### 3.1.2 Study of Fe^2+^ immobilization ability of enriched microorganisms

**Figure 3:**
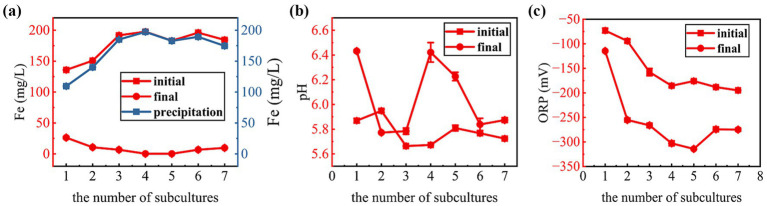
Changes in supernatant Fe concentration **(a)**, pH **(b)**, and ORP **(c)** during the transfer enrichment process.

Sulfate-reducing functional microorganisms can reduce sulfate to sulfur-negative ions (S^2−^), and sulfur-negative ions can react with Fe^2+^ in the medium to form an insoluble black FeS immobilization product, Fe^2+^ immobilization ability can reflect the enrichment effect of sulfate-reducing functional microorganisms. The Fe^2+^ immobilization efficiency is presented in Figure 3a. During the transfer incubation process, the Fe^2+^ immobilization rate basically increased with the increase of the number of transfers, and the immobilization rate in the first two passages remained below 50%, but by the fourth transfer, the immobilization rate increased significantly to more than 90% and remained essentially stable in subsequent transfers (4th to 7th). This indicates a gradual enrichment of SRBs and enhanced sulfate reduction activity. This result suggests that the SRB community became more dominant and metabolically active after several rounds of passaging culture, indicating that the repeated passaging process enriched SRBs adapted to the medium and culture conditions.

#### 3.1.3 pH and ORP changes

Measurements of pH and oxidation–reduction potential (ORP) in the culture supernatant provided further validation of SRB activity. Sulfate reduction typically consumes protons, leading to a pH increase, and creates a reducing environment, resulting in an ORP decrease (Zhang M. et al., 2022a). As shown in Figure 3b, the final pH values for each transfer cycle (except the second) were elevated compared to their initial values, ranging from 5.8 to 6.4. This increase in pH is a typical result of sulfate reduction by SRBs, as the sulfate reduction process depletes protons (H^+^) and produces hydroxide ions (OH^−^), thus leading to alkalinization of the medium (Novair et al., 2024). The slight decrease in pH observed in the second transfer may be attributed to the fact that sulfate-reducing microorganisms had not yet become the dominant population at the time of the second transfer, and acid-producing microorganisms temporarily dominated, and then sulfate-reducing bacteria gradually became the dominant population as the enrichment process progressed (Zhang et al., 2017). The ORP values demonstrated a consistent downward trend throughout the transfer process (Figure 3c), decreasing from approximately −100 mV at the start of the first transfer to below −250 mV after the second transfer, and subsequently stabilizing at this low level. Lower ORP values imply a stronger reducing atmosphere, which favors the growth and metabolic activity of anaerobic bacterial SRB. The stable low ORP value after the second transfer indicated that the culture system had established stable anaerobic conditions, which also promoted the dominance of SRB.

### 3.2 Study on the effect of nutrient factor concentration on the sulfate reduction metabolic performance of SRB

**Figure 4:**
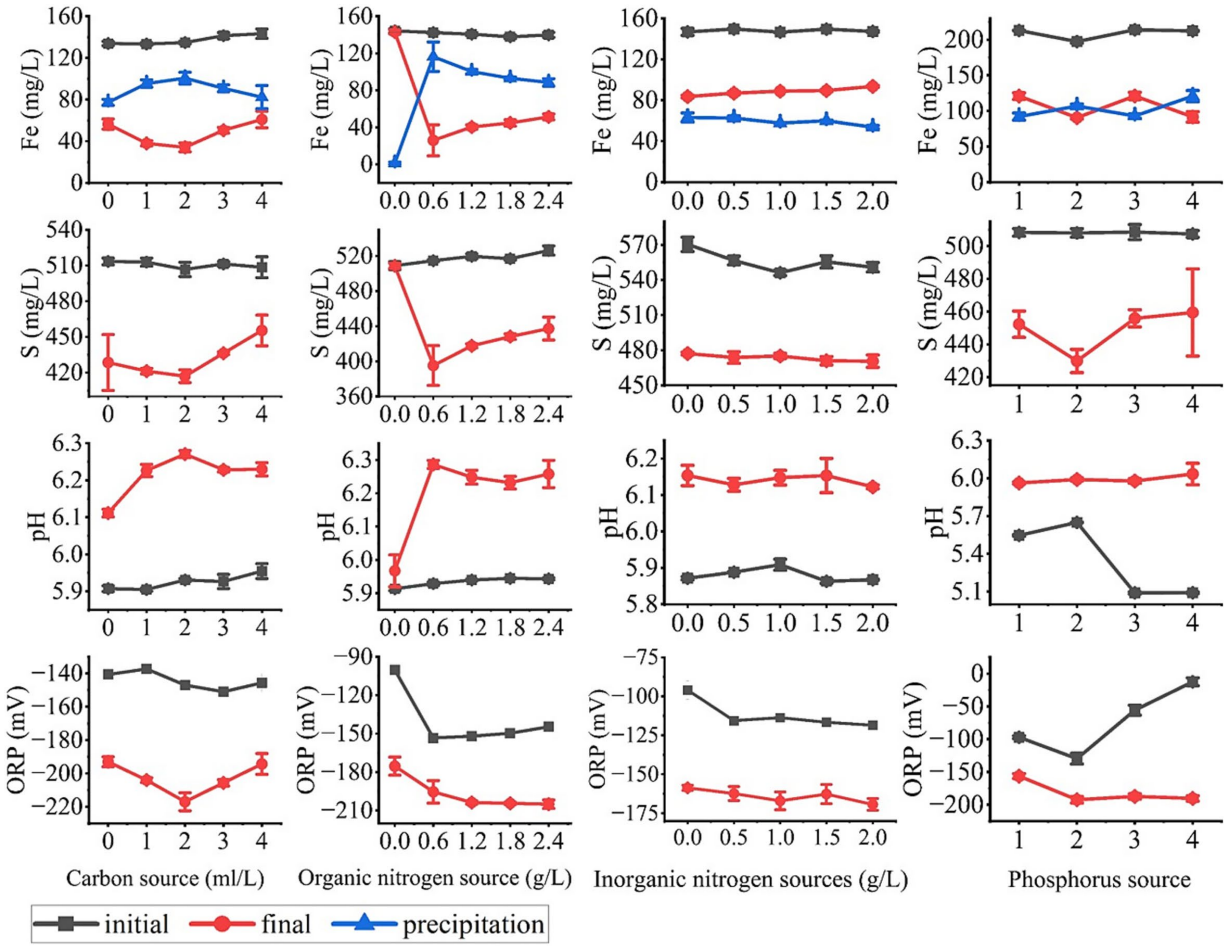
Effects of nutrient factor concentrations on the metabolic performance of sulfate reduction of SRBs Changes in supernatant soluble Fe, S concentrations, pH and ORP values in the studied experiments.

As a chemoheterotrophic microorganism, the concentration of nutrient factors is the key to regulate the metabolic activity of SRB. Although Postgate medium is commonly used for SRB screening (Labastida et al., 2024), the indigenous SRB isolated from Antimony mine have different nutrient factor requirements than conventional strains due to the complex pollution environment. In view of its mine specificity and application scenarios of multiple heavy metal stresses, it is necessary to optimize the concentration of nutrient factors in the medium for the metabolic characteristics of this colony under compound pollution to maximize its function in the immobilization of antimony and arsenic and other heavy metals. This section investigates the individual effects of carbon source (sodium lactate), organic nitrogen source (yeast extract), inorganic nitrogen source (ammonium chloride), and phosphorus source type/concentration on sulfate reduction by SRB. While keeping other nutrient factors constant, we varied the target nutrient and assessed its impact by measuring soluble Fe/S concentrations, pH, and ORP in the supernatant (Figure 4).

#### 3.2.1 Carbon source

SRBs can utilize over 100 organic compounds as electron donors, and lactate has been widely documented as the preferred electron donor for supporting the growth and metabolic demands of diverse SRBs, exhibiting distinct advantages in promoting SRB proliferation and metabolic activity (Teclu et al., 2009; Costa et al., 2014). The influence of sodium lactate concentration on Fe^2+^ immobilization efficiency and total sulfur precipitation in the solution is presented in Figure 4, where a concentration of 2.0 mL/L resulted in the highest Fe^2+^ immobilization rate (>95%) and total sulfur precipitation, significantly outperforming both lower and higher concentrations.

At lower sodium lactate concentrations (0–1.0 mL/L), the carbon source was insufficient to sustain optimal SRB growth and metabolism, resulting in diminished sulfide production and consequently lower Fe^2+^ immobilization. Conversely, at higher concentrations (3.0–4.0 mL/L), excess carbon sources likely induced imbalances in the medium’s redox potential or osmotic pressure, thereby inhibiting SRB activity. pH and ORP measurements further corroborate these observations: the 2.0 mL/L sodium lactate treatment showed the highest final pH and lowest final ORP, which are closely associated with the sulfate reduction reactions mediated by SRBs.

#### 3.2.2 Organic nitrogen sources

Yeast extract is a commonly used organic nitrogen source in SRB culture (Chanda et al., 2020). Figure 4 illustrates that a yeast extract concentration of 0.6 g/L achieved the maximum iron immobilization rate. In the group without yeast extract addition, no obvious black precipitates were observed in the reaction flasks after 7 days of incubation, indirectly suggesting minimal SRB growth. This is further supported by the pH values, which remained nearly unchanged in the yeast extract-free group. This phenomenon is likely because yeast extract contains essential components for SRB growth, such as amino acids, vitamins, and growth factors, making it indispensable for SRB proliferation and metabolism (Zhang et al., 2017). Conversely, the efficiency of Fe immobilization gradually decreased with increasing yeast extract concentrations, indicating that supra-optimal levels also inhibited SRB growth and metabolic activity. This inhibition is potentially attributed to the accumulation of toxic by-products from yeast extract degradation or an imbalance in the medium’s carbon-to-nitrogen ratio (Zhang et al., 2017). Additionally, pH and ORP measurements showed more pronounced changes in the 0.6 g/L yeast extract treatment, further confirming this concentration as the optimal choice in the present study.

#### 3.2.3 Inorganic nitrogen sources

Ammonium chloride is a widely used inorganic nitrogen source in microbial cultures but has been less frequently employed in cultivating sulfate-reducing bioremediation consortia. As shown in Figure 4, Fe^2+^ immobilization efficiency decreased with increasing NH_4_Cl concentration, with the highest efficiency observed in the absence of NH_4_Cl. This observation aligns with previous studies reporting that inorganic nitrogen inhibits SRB growth, potentially due to the toxicity of high ammonium ion (NH_4_^+^) concentrations or competition with other nutrients (Hasanzade et al., 2019). pH and ORP changes further reflected this inhibitory effect: these parameters showed less pronounced variations in the presence of NH_4_Cl, indicating reduced metabolic activity of SRBs. Collectively, these results indicate that inorganic nitrogen is not essential for the growth of the enriched SRBs and may even be detrimental, suggesting that organic nitrogen sources alone are sufficient for SRB cultivation.

#### 3.2.4 Phosphorus sources

Both K_2_HPO_4_·3H₂O and KH_2_PO_4_ are common phosphorus sources in microbial cultures, but they differ in their dissociation equilibria. Excessive PO_4_^3−^ can directly precipitate with ions such as Fe^2+^, Ca^2+^, and Mg^2+^ in the system, limiting microbial utilization. Thus, the type and concentration of phosphorus sources significantly affect SRB performance. This section investigates whether the selection and concentration of phosphate sources adversely impact SRB growth and metabolism. As shown in Figure 4, SRB activity was lower in the P1 and P3 groups, likely due to insufficient phosphorus supply. The P4 group, supplemented with 0.5 g/L KH_2_PO_4_, exhibited relatively high Fe precipitation; however, elemental sulfur precipitation was relatively low. This suggests that efficient Fe precipitation in the P4 group was not solely dependent on SRB growth and metabolism. Combined with comparisons of total phosphorus concentrations across the four treatments, it is evident that Fe precipitation was positively correlated with the initial phosphorus concentration in the system. High phosphate concentrations may have precipitated with metal ions, reducing their availability for microbial growth. Additionally, initial samples in the P4 group exhibited a lower pH and higher ORP, which are detrimental to SRB proliferation and the remediation of heavy metal-contaminated sites. The P2 treatment (0.50 g/L K_2_HPO_4_·3H₂O) demonstrated the best overall performance, achieving high Fe^2+^ immobilization efficiency and total sulfur precipitation. These results indicate that K_2_HPO_4_·3H₂O is a more suitable phosphorus source for SRB enrichment, and a concentration of 0.50 g/L achieves a balance between providing sufficient phosphorus and avoiding metal ion precipitation.

#### 3.2.5 Growth kinetics of optimized SRB

The growth of microorganisms is directly linked to their ability to efficiently exert remediation functions. Investigating the temporal variations of physicochemical parameters during microbial growth constitutes a primary approach to understanding microbial proliferation. Under optimized culture conditions (2.0 mL/L sodium lactate, 0.6 g/L yeast extract, 0 g/L NH_4_Cl, and 0.50 g/L K_2_HPO_4_·3H₂O), a systematic study on the growth kinetics of sulfate-reducing bacteria (SRB) was conducted over a 16-day period.

**Figure 5:**
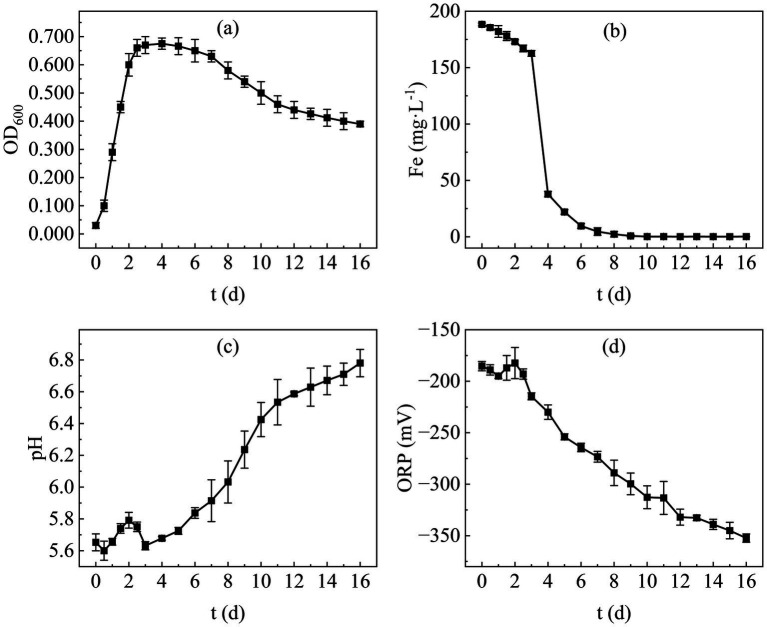
Growth kinetics of SRB in **(a)** OD_600_; **(b)** Fe^2+^; **(c)** pH; **(d)** ORP.

The optical density at 600 nm (OD_600_) is a key parameter for monitoring biomass changes, and its temporal progression defines the microbial growth curve. Figure 5a presents the growth curve of the bioremediation microbial consortium after enrichment optimization, revealing that the population enters the logarithmic growth phase within 3–4 days. Post the 4th day, the bacterial concentration gradually declines, indicating a reduction in the biomass of the mixed microbial community. Despite the late-stage decrease in OD_600_, the decline rate is slow, with a value of 0.390 still observed on day 16 equivalent to 57.7% of the maximum OD_600_(0.675). By the termination of the assay, no significant death was noted. This growth pattern suggests that the optimized SRB exhibit a short lag phase and a relatively prolonged stationary phase, enabling them to sustain metabolic activity over an extended period and rendering them suitable for long-term remediation of heavy metal-contaminated sites.

Temporal changes in total Fe concentration are illustrated in Figure 5b. It is evident that the total Fe concentration in the supernatant decreased slowly within the first 3 days post-inoculation, followed by a rapid decline during the 3–7 day period. From day 7 onwards, the concentration decreased gradually and approached zero. This trend indicates that the sulfate reduction rate of the remediation microbial consortium was not constant after inoculation; competition among diverse pre-existing functional microorganisms occurred in the early stage, and SRB could only function effectively once their abundance reached a threshold level.

Variations in pH and ORP further confirmed the favorable growth of SRB. Figures 5c,d depict the temporal dynamics of pH and ORP, respectively. Both parameters fluctuated to a certain extent within the first 3 days, potentially attributed to intense inter-population competition. After day 3, however, the pH increased continuously, reaching approximately 6.8 by day 16. Concurrently, the ORP decreased steadily from day 3 onwards, dropping below −350 mV by day 16, indicating that SRB became the dominant population within the remediation consortium from day 3 onwards.

### 3.3 Microbial immobilization study of complex pollutants in antimony mine wastewater

#### 3.3.1 Immobilization rate of heavy metal(loid)s

**Figure 6:**
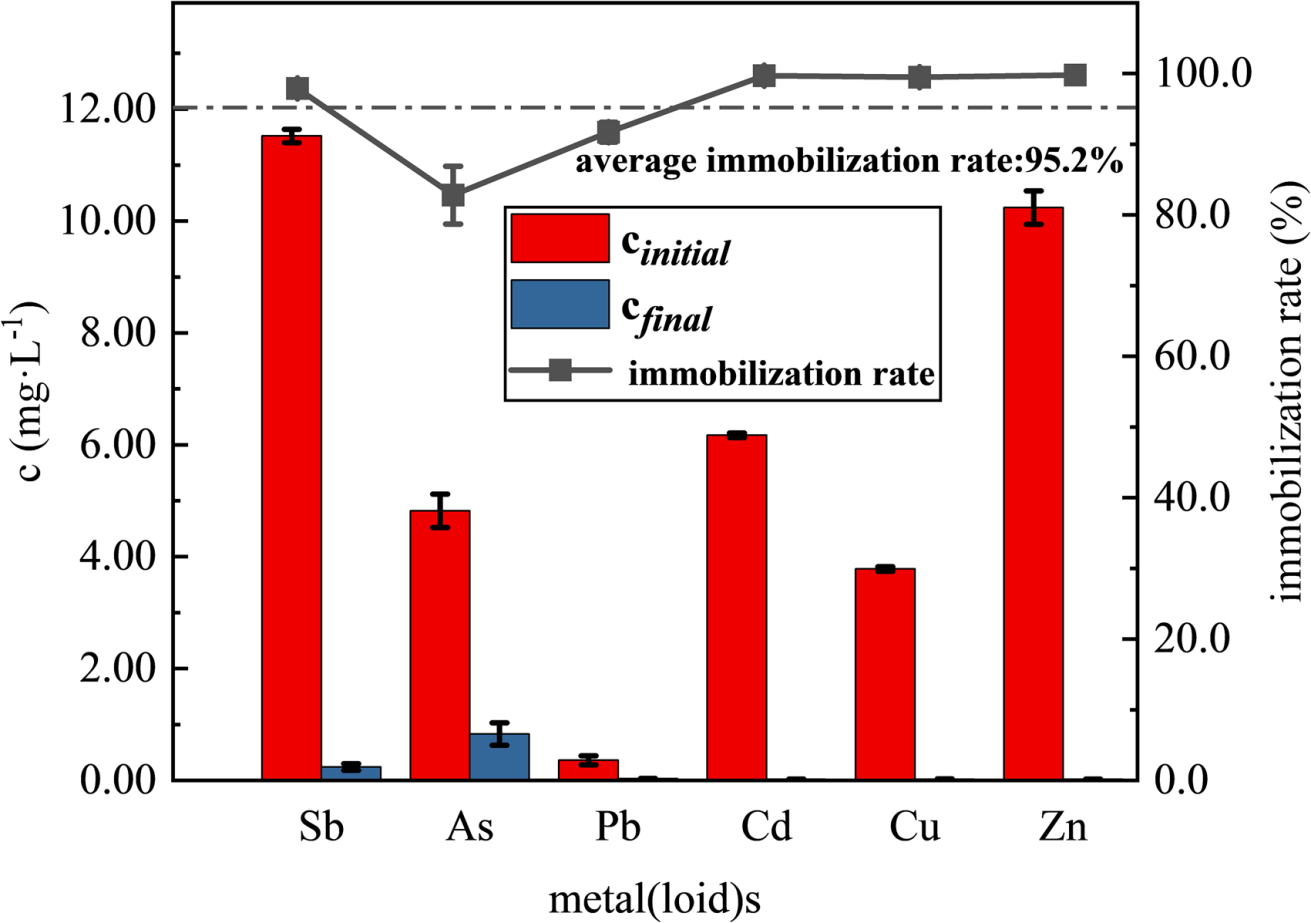
Remediation of simulated heavy metal(loid)s contaminated wastewater from antimony mining area by SRB.

After SRB addition and reaction for 7d, significant reddish brown precipitation was observed in all three treatment groups. It was found that the optimized SRB showed higher immobilization rate in immobilization most of the heavy metals in the simulated wastewater by examining the concentrations of various elements in the supernatant, the results of which are shown in Figure 6. As shown in Figure 6, the concentrations of various heavy metals were drastically reduced with an average immobilization rate of 95.2%. Among them, the concentrations of Cd, Cu and Zn in the supernatant were reduced to below the detection limit, and the immobilization rate reached 99.7, 99.5 and 99.8%, respectively, and Pb also had a better immobilization effect, with an immobilization rate of 91.7%. The average concentration of Sb was reduced to 0.24 mg/L, and the immobilization rate was about 97.9%; the concentration of As was only reduced to 0.83 mg/L, with an immobilization rate of about 82.8%, which is lower than those of the remaining five heavy metal(loid)s. Compared with recent studies, the SRB consortium in this work achieved higher Sb immobilization efficiency (97.9%) than that reported by Zhang et al. (2023) (80.2% at initial Sb of 10 mg/L), and maintained good performance even under the coexistence of multiple heavy metals, demonstrating its excellent adaptability to complex pollution in antimony mining areas. Similarly, the immobilization rate of As (82.8%) is comparable to or higher than those reported in other studies using SRB for arsenic removal (Alam and McPhedran, 2019; Yang et al., 2024).

As reported in the literature, achieving the desired level of arsenic remediation with SRB is often challenging (Alam and McPhedran, 2019). The primary mechanism for heavy metal immobilization by SRB is the production of sulfide (S^2−^) through dissimilatory sulfate reduction. The S^2−^ ions react with cationic heavy metal ions to form highly insoluble metal sulfides, characterized by very low solubility products (*K*_sp_), which constitutes the principal immobilization pathway for these cations. The three groups of treatments produced significant reddish-brown precipitates which is usually due to the formation of sulfides of heavy metals such as Sb_2_S_3_, As_2_S_3_, and ZnS (Yang et al., 2024; Zhuang et al., 2025). The low solubility products (*K*_sp_) of the corresponding metal sulfides (e.g., Sb_2_S_3_: ~1.6 × 10^−98^, PbS: 8.0 × 10^−28^, CdS: ~8.0 × 10^−27^, CuS: ~6.3 × 10^−36^, ZnS: ~2.0 × 10^−22^) (Liu et al., 2018; Mafane et al., 2025; Marques and Rodrigues, 2025) explain the high immobilization rates observed for these cationic metals in the simulated wastewater. It is noteworthy that antimony showed a measurable residual concentration (up to 0.24 mg/L), which appears inconsistent with the extremely low *K*_sp_ of Sb_2_S_3_. This discrepancy can be attributed to the formation of its thioantimony (Ye et al., 2019). The relatively lower immobilization efficiency of arsenic is attributed not only to the higher solubility of its sulfides (Liu et al., 2018) but also to the formation of soluble thioarsenic complexes under reducing, sulfidic conditions (Gao et al., 2021).

#### 3.3.2 Characterization of immobilization products

In order to investigate the mechanism of SRB remediation of heavy metal(loid)s pollutants at various levels, SEM-EDS, XRD and FT-IR characterization of the immobilization products was carried out (Ma et al., 2023).

**Figure 7:**
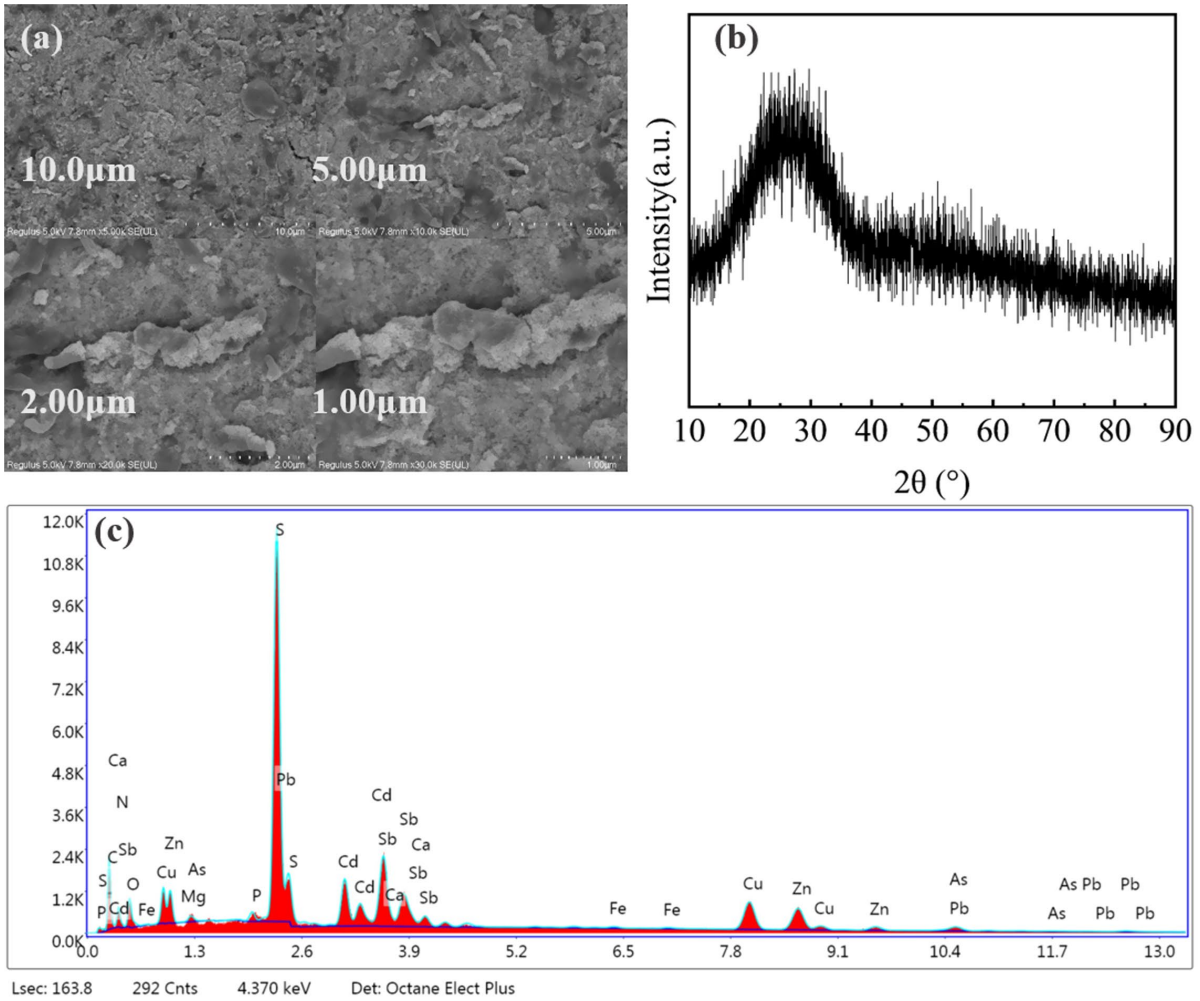
Characterization of the immobilization products by SEM, XRD, and EDS: **(a)** SEM image showing the pervasive fine-grained morphology of the precipitates; **(b)** XRD pattern; **(c)** EDS spectrum.

Insight into the mechanism of heavy metal immobilization was provided by SEM-EDS analysis of the immobilized products. SEM images (Figure 7a) showed the presence of fine-grained precipitates with a non-homogeneous phase structure, which is typical for the formation of metal sulfides. The immobilization products were analyzed by X-ray powder diffraction (XRD) and the results are shown in Figure 7b. The XRD analysis, on the other hand, revealed that no obvious diffraction peaks appeared, which indicates that the immobilization products formed were mostly in an amorphous form (Kocar et al., 2010). The EDS analysis (Figure 7c) confirmed the presence of S, O, and all target heavy metals in the precipitates. The high abundance of sulfur suggests that metal sulfides were a major component of the immobilization products.

EDS analysis detected significant amounts of C, N and O in the immobilized products, which are characteristic elements of microbial cells. Microbial cells can adsorb heavy metals through functional groups on the cell surface, such as carboxyl, amino, and phosphate groups (Chen et al., 2025), which suggests that in addition to sulfide precipitation, biosorption by SRB cells plays a role in immobilization heavy metals.

**Figure 8:**
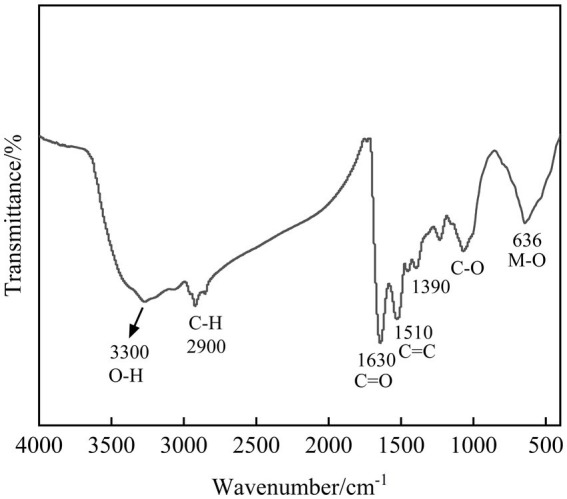
FT-IR pattern of products of SRB immobilization of composite pollutants.

Fourier transform infrared (FT-IR) spectroscopy was employed to identify functional groups in the immobilization products, providing insights into the molecular-level mechanisms involved in the metal immobilization process. The FT-IR spectrum of the SRB-generated immobilization products is presented in Figure 8. The broad absorption band around 3,300 cm^−1^ is characteristic of O-H stretching vibrations, potentially indicating the presence of metal hydroxides (Das et al., 2012). The metabolic activity of SRB elevates the pH, facilitating the precipitation of some heavy metals as insoluble hydroxides. The weak bands near 2,900 cm^−1^, assigned to C-H stretching vibrations (Miao et al., 2018), originate from aliphatic hydrocarbons in microbial cells and their secreted extracellular polymeric substances (EPS). These extracellular polymers can bind heavy metal ions, contributing to immobilization and mitigating metal toxicity to the microbial cells. The characteristic peak at 1630 cm^−1^ is generally a stretching vibration of C=O (Di et al., 2022), which tends to complex, suggesting that the microbial immobilization process may be accompanied by the occurrence of complexation reactions and the formation of some heavy metal complexes. The peak near 1,500 cm^−1^ is located in the double bond region, which is generally a vibration of the C=C double bond (Zhang et al., 2023), and the peaks here may be related to microbes themselves or secreted organic matter as well. The absorption features in the 1,200 ~ 1,000 cm^−1^ range, attributable to C-O stretching vibrations (Lv et al., 2022), suggest the presence of oxygen-containing functional groups. Considering the experimental conditions, the formation of metal carbonates is plausible. The range of 675 ~ 477 cm^−1^ belongs to the low-frequency region, and the peaks here are generally related to the vibration modes of the metal-oxygen bond (Zhang et al., 2018), and it can be presumed that the immobilization product contains more metal oxides. Overall, the products of microbial immobilization of heavy metals may contain a variety of components such as heavy metal hydroxides, carbonates, metal oxides and complexes, rather than simple sulfide precipitation. This mechanism is particularly important for metals like As, which could explain why they are much less efficient at sulfide precipitation but still exhibit an 82.8% immobilization rate. The combined effect of sulfide precipitation and biosorption enhances the overall immobilization ability of SRBs, especially for metals that form less stable sulfides.

### 3.4 Bioremediation study of antimony tailings composite pollution by SRB

#### 3.4.1 Study on the change of tailings heavy metal concentration with remediation time during the bioremediation process

**Figure 9:**
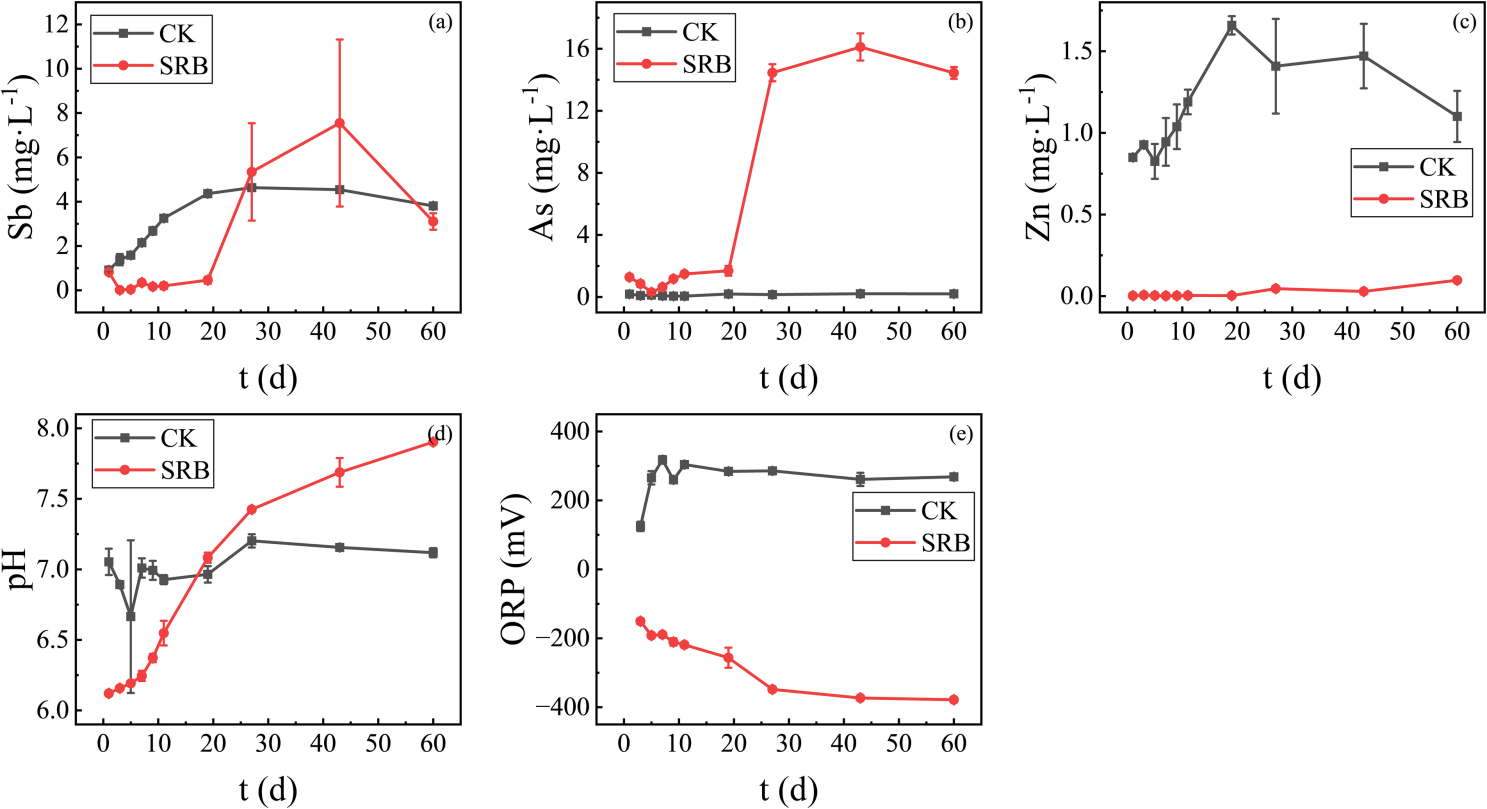
Variation of various heavy metal concentrations and pH and ORP values during SRB remediation of antimony tailings **(a)** Sb; **(b)** As; **(c)** Zn; **(d)** pH; **(e)** ORP.

The antimony tailings were remediated using SRB obtained after enrichment and optimization of nutrient factors, and the remediation effect was obtained as shown in Figure 9. As can be seen from Figure 9, SRB showed significantly different results for Sb, As, and Zn. In the process of SRB remediation, the concentration of Sb decreased significantly in the first 20 days, then increased rapidly and exceeded the concentration of the control group after 20 days. The concentration of As in the remediation group was higher than that of the control group at the beginning of the experiment and even more strongly resolubilized after 20 days. The observed re-solubilization of Sb and As is likely due to the formation of soluble thioanionic complexes (e.g., SbS_3_^3−^, AsS_3_^3−^) under the alkaline and highly reducing conditions generated by SRB metabolism (Ye et al., 2019; Gao et al., 2021). The concentration of Zn has been maintained at much lower concentrations since the addition of SRB with good remediation. This suggests that Zn may be effectively immobilized by SRB through the formation of ZnS precipitates (Yan et al., 2023).

The pH and ORP of the remediation system affected by SRB metabolism significantly influenced the remediation of heavy metals. During the remediation process, the pH and ORP of the supernatant of the remediation system were tracked and measured, and their variations are shown in Figures 9d,e. As can be seen from the figures, the pH value of the control group basically remained stable, while the pH value of the SRB remediation group climbed up, from about 6.0 to nearly 8.0 (Figure 9d), which indicates that the metabolism of SRBs in this remediation system functions well. In addition to sulfides, the rise in pH can also promote the precipitation of metal hydroxides. The results of redox potential measurements also showed that the SRB remediation group maintained a better reducing atmosphere during the nearly 60 d of remediation, decreasing to below −300 mV in the later stages, while the control group remained in an oxidizing atmosphere. SRB metabolism generates S^2−^ and raises pH, exerting dual (positive and negative) effects on the immobilization of Sb and As in antimony tailings. On the one hand, it promotes the formation of Sb and As sulfides and facilitates the solidification of Sb and As released via acid dissolution. On the other hand, it also induces the reduction and dissolution of Sb and As, as well as the formation of thiol compounds. This presents unique challenges for remediating composite pollution in antimony mining regions.

#### 3.4.2 Study on the influence of sulfate concentration on the bioremediation effect of antimony tailings

**Figure 10:**
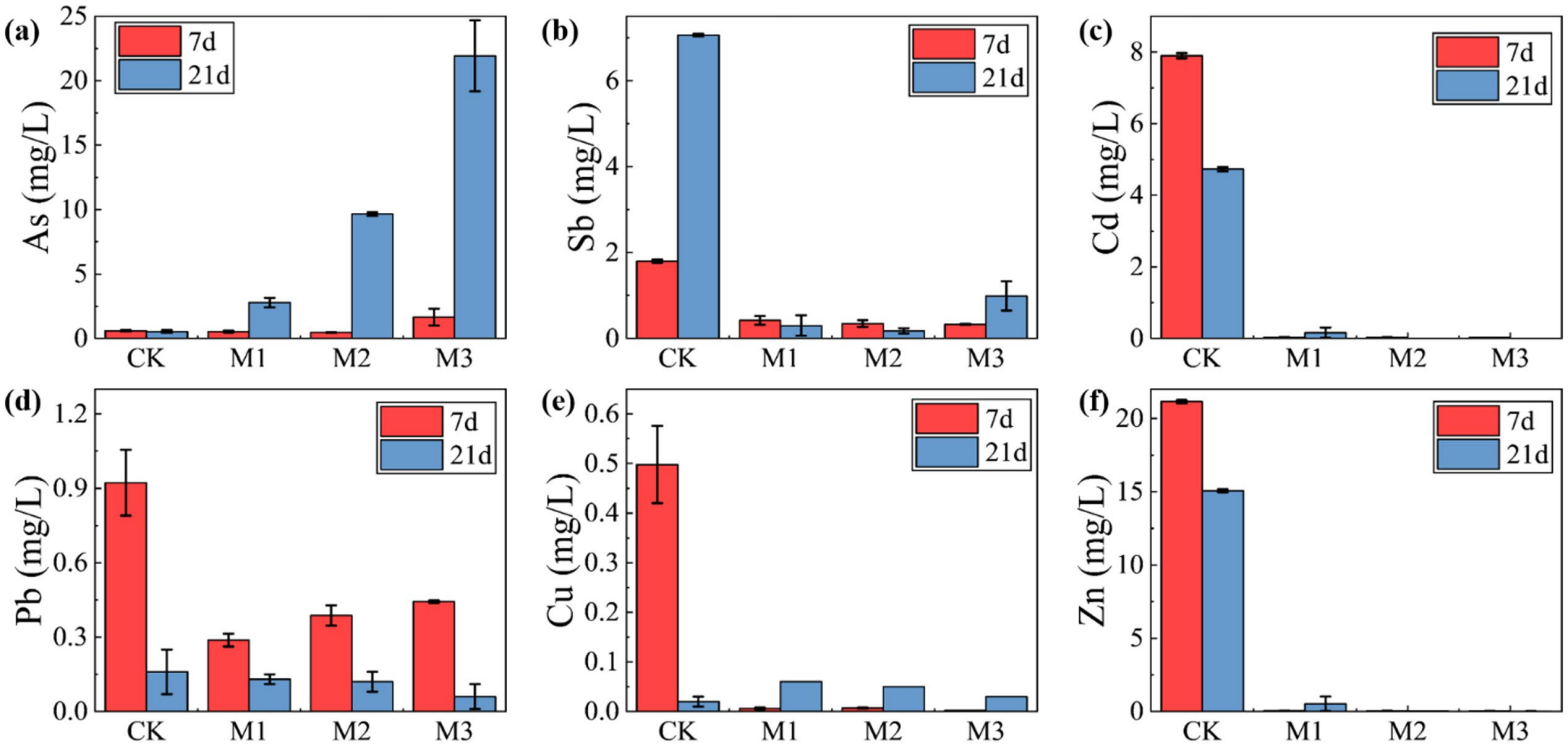
Variation of heavy metal concentration with sulfate concentration in antimony tailings supernatant: **(a)** As; **(b)** Sb; **(c)** Cd; **(d)** Pb; **(e)** Cu; **(f)** Zn.

The influence of sulfate concentration on the immobilization of heavy metals and metalloids in antimony tailings was investigated by adjusting the initial concentration of MgSO_4_·7H_2_O in the SRB-tailings system. The variations in supernatant concentrations of the target pollutants after treatment are summarized in Figure 10. A clear dichotomy was observed between the behaviors of cationic heavy metals and metalloids. The concentrations of cationic heavy metals, including Cu, Cd, and Zn, were effectively reduced and maintained at low levels across all SRB-amended treatments (M1, M2, and M3), demonstrating successful immobilization irrespective of the applied sulfate concentration. In stark contrast, the immobilization efficacy for the metalloids Sb and As was highly dependent on sulfate concentration. For arsenic (As), a significant re-solubilization phenomenon was observed, particularly in the M3 group (4.0 g/L sulfate), where its final concentration in the supernatant exceeded that of the control group (CK). This re-solubilization was substantially suppressed in the M1 group (1.0 g/L sulfate). Antimony (Sb) exhibited a similar trend, with the most effective immobilization achieved in the M2 group (2.0 g/L sulfate), while higher sulfate levels (M3) promoted its release.

This marked difference in behavior can be attributed to fundamentally distinct immobilization mechanisms. The consistent and effective immobilization of cationic metals (Cu, Cd, Zn) is primarily governed by the precipitation of highly insoluble metal sulfides (e.g., CuS, CdS, ZnS) upon reaction with biogenic S^2−^(Zhang Z. et al., 2022b). The very low solubility products (*K*_sp_) of these sulfides ensure robust immobilization across a wide range of sulfate concentrations. Conversely, the fate of arsenic is more complex and less favorable. As an oxyanion, it does not form stable sulfide precipitates as readily as cationic metals (Alam and McPhedran, 2019). Under the strongly reducing and sulfidic conditions generated by active SRB metabolism, As (V) can be reduced to As(III), which not only is more mobile but can also form a series of soluble thioarsenic complexes (e.g., AsS_3_^3−^, (As(III)S_3_)^3−^) in the presence of excess sulfide (Gao et al., 2021; Yang et al., 2022). This secondary reaction pathway competes with any potential precipitation process, leading to the re-solubilization observed at high sulfate/sulfide levels (M3). A analogous mechanism involving the formation of soluble thioantimonate complexes is likely responsible for the sulfate-dependent re-solubilization of Sb (Ye et al., 2019; He et al., 2020).

In conclusion, these findings demonstrate that sulfate concentration is a critical lever controlling the remediation outcome. While it is essential for powering the SRB metabolism that immobilizes cationic metals, it can simultaneously trigger the re-mobilization of metalloids through soluble thio-complex formation. Therefore, optimizing the sulfate dosage is imperative to strike a balance between achieving high immobilization efficiency for cationic heavy metals and minimizing the leaching risk of metalloids like As and Sb in co-contaminated systems.

## 4 Conclusion

This study successfully developed a bioremediation strategy using enriched indigenous sulfate-reducing bacteria (SRB) to address the complex heavy metal pollution in antimony mining areas. Through systematic enrichment and optimization processes, we obtained a highly active SRB consortium dominated by the genus *Clostridium*. The optimal growth medium was determined to contain 2.0 mL/L sodium lactate as carbon source, 0.6 g/L yeast extract as organic nitrogen source, and 0.50 g/L K₂HPO₄·3H₂O as phosphorus source, which significantly enhanced the metabolic activity and sulfate reduction capability of the bacterial community. In simulated wastewater treatment experiments, this optimized SRB system demonstrated remarkable immobilization performance, achieving removal rates exceeding 97.9% for antimony, lead, cadmium, copper, and zinc, while arsenic was immobilized at 82.8%. The remediation effectiveness was further verified in actual antimony tailings, where SRB consistently maintained high immobilization efficiency for cationic heavy metals including zinc, cadmium, copper, and lead throughout the treatment period. However, the treatment of metalloids presented a more complex scenario, with antimony and arsenic showing significant redissolution behavior. This challenge was effectively addressed by implementing a sulfate concentration control strategy, which substantially suppressed the remobilization of these problematic metalloids. The key innovations of this study include: (1) successful enrichment of indigenous SRB from antimony tailings with high adaptability; (2) systematic optimization of nutrient factors to enhance SRB metabolic activity in composite pollution systems; (3) revealing the influence of sulfate concentration on Sb/As redissolution and proposing a regulatory strategy; and (4) achieving synergistic immobilization of Sb, As, and multiple heavy metals, offering new insights for remediation of complex mining pollution. These findings establish a solid scientific foundation for scaling up SRB mediated bioremediation technologies in mining environments. From a technological economic perspective, the SRB-based bioremediation strategy developed herein holds promise for cost-effective application. The primary nutrients (sodium lactate, yeast extract, phosphate) are commercially available and relatively inexpensive. The most significant potential cost savings lie in the utilization of indigenous microorganisms, which reduces the need for specialized, non-native bacterial consortia and enhances system resilience. Future research directions should focus on field scale validation of this technology, particularly through the development of immobilized microbial formulations for enhanced stability and longevity in practical applications. Additionally, the resource recovery potential of the metal sulfide rich precipitates generated during the wastewater remediation process warrants further investigation, potentially opening pathways for integrating environmental remediation with valuable metal recovery in a circular economy framework.

## Data Availability

The raw data supporting the conclusions of this article will be made available by the authors, without undue reservation.
